# CD8^+^ T-Cell Responses before and after Structured Treatment Interruption in Ugandan Adults Who Initiated ART with CD4^+^ T Cells <200 Cell/*μ*L: The DART Trial STI Substudy

**DOI:** 10.1155/2011/875028

**Published:** 2011-01-18

**Authors:** Jennifer Serwanga, Susan Mugaba, Auma Betty, Edward Pimego, Sarah Walker, Paula Munderi, Charles Gilks, Frances Gotch, Heiner Grosskurth, Pontiano Kaleebu

**Affiliations:** ^1^MRC/UVRI Uganda Research Unit on AIDS, 51-59 Nakiwogo Road, Entebbe, Uganda; ^2^MRC Clinical Trials Unit, 222 Euston Road, London NW1 2DA, UK; ^3^Imperial College London, South Kensington Campus, London SW7 2AZ, UK; ^4^Department of Immunology, Imperial College, Chelsea and Westminster Hospital, London SW10 9NH, UK; ^5^London School of Hygiene & Tropical Medicine, University of London, London WC1E 7HT, UK

## Abstract

*Objective*. To better understand attributes of ART-associated HIV-induced T-cell responses that might be therapeutically harnessed. *Methods*. CD8^+^ T-cell responses were evaluated in some HIV-1 chronically infected participants of the fixed duration STI substudy of the DART trial. Magnitudes, breadths, and functionality of IFN-*γ* and Perforin responses were compared in STI (*n* = 42) and continuous treatment (CT) (*n* = 46) before and after a single STI cycle when the DART STI trial was stopped early due to inferior clinical outcome in STI participants. *Results*. STI and CT had comparable magnitudes and breadths of monofunctional CD8^+^IFN*γ* ^+^ and CD8^+^Perforin^+^ responses. However, STI was associated with significant decline in breadth of bi-functional (CD8^+^IFN*γ* ^+^Perforin^+^) responses; *P* = .02, Mann-Whitney test. *Conclusions*. STI in individuals initiated onto ART at <200 CD4^+^ T-cell counts/*μ*l significantly reduced occurrence of bifunctional CD8^+^IFN*γ* ^+^/Perforin^+^ responses. These data add to others that found no evidence to support STI as a strategy to improve HIV-specific immunity during ART.

## 1. Introduction

Previous studies correlated ART uptake with diminution of HIV-specific responses [1–3], whilst others linked it with restoration of these responses [4–6]. Uptake of ART has also presented challenges such as high pill burden, drug resistance, cost, and drug-induced toxicities. Structured treatment interruption has been widely evaluated in an effort to decrease costs and side effects of ART uptake and to investigate associated immunological and clinical outcomes. Consequently, several large clinical trials have explored whether minimizing dose [7–10], or duration of ART exposure [11, 12] and therefore, their toxic effects would be beneficial.

The cellular arm of the immune system has been associated with protection from HIV disease progression [13]. This has been remarkably demonstrated in CD8^+^ T-cell depletion studies in macaque models [14–16] as well as in acute infection studies correlating the emergence of virus specific CD8^+^ T cells with control of viraemia [17]. In addition, correlation between slow HIV-1 disease progression and protective HLA allele-induced CTL responses has been observed [18], and associations between viral escape in targeted HIV epitopes and elevation of plasma viral loads have been demonstrated [19]. Despite this body of evidence, consistent quantitative and qualitative correlates of protection remain elusive. Evaluations comparing breadths and magnitudes of CD8^+^ T-cell responses in infected persons have sometimes failed to show any association between these parameters and viral load [18, 20, 21]. Moreover, despite the preservation and increase in HIV-specific CD8^+^ T-cell responses in individuals who initiated ART during acute infection [22, 23], attempts to boost T-cell responses, for example, through autovaccination during ART interruptions in chronic HIV infection, have proven disappointing [9, 19, 24–26].

The potential for STI to boost HIV-specific immunity through controlled autologous virus exposure has been previously evaluated in chronically infected HIV patients [27–29] and has been reviewed in [30]. In some of these studies, CD8^+^ T-cell responses were boosted by re-exposure to autologous virus, although increases in virus-induced CD4^+^ T-cell responses were transient. Marked interpatient heterogeneity and small cohort sizes in previous studies yielded inconclusive and inconsistent results [26, 31–33]. While functional attributes of CD8^+^ T-cell responses have accounted for differential disease outcomes observed in chronic HIV infection [34–36], the extents to which differences in CD8^+^ T-cell functionality occur during STI remain unclear. 

In this study, we evaluated a proportion of DART trial participants [37] who enrolled into the DART STI substudy [38] in order to better understand the possible immunological outcomes of STI in individuals initiated onto ART with advanced HIV disease (<200 CD4^+^ T cells/*μ*L). Participants randomized to STI or to continuous treatment (CT) at one clinical centre (MRC/UVRI, Entebbe, Uganda) were compared in order to reevaluate the hypothesis that STI would allow regeneration of HIV-specific responses through cyclical viral antigen exposure whilst CT would not allow viral antigen exposure and consequent regeneration of immune responses.

## 2. Methods

### 2.1. Study Design and Population

The multisite DART main trial (ISCRTN 13968779-DART) [37] recruited 3316 chronically HIV-infected, ART-naïve adults (except for ART exposure during pregnancy for prevention of mother-to-child transmission) with WHO stage 2, stage 3, or stage 4 symptomatic disease and CD4^+^ counts ≤200 cells/*μ*L at screening to primarily compare clinical driven monitoring with laboratory monitoring plus clinical monitoring as strategies of ART delivery. A fixed duration STI randomization substudy (12 weeks on ART and 12 weeks off ART per STI cycle) was nested within the DART trial to primarily evaluate whether STI was clinically noninferior to continuous treatment. Participants, who had attained ≥300 CD4^+^ T cells/*μ*L by 48 or 72 weeks after DART trial entry, underwent a second randomization at 52 or 76 weeks to either STI (*n* = 408) or CT (*n* = 405), with intention to follow up 8 STI cycles. At the time of STI/CT randomization, we consecutively recruited 60 STI and 60 CT participants from each arm. Heparinised blood (10 mls) was collected at the beginning and end of one STI cycle corresponding to week 0 and week 12, respectively. By the time the trial was stopped early following a DSMC review, 42 STI and 46 CT participants had completed one full STI cycle. None of the 88 evaluated subjects (60 women) received any prior exposure to ART even for prevention of mother-to-child transmission.

### 2.2. CD4^+^ T-Cell Count Quantification and Timing of T-Cell Response Evaluations

Scheduled 12 weekly CD4^+^ T-cell counts were performed within the main DART trial using FACScount (Becton Dickinson) according to manufacturer's protocols. The beginning of the first STI/CT randomization cycle was timed to occur 4 weeks after the last prerandomization CD4^+^ count. The next CD4^+^ count occurred 8 weeks after the start of the cycle and 8 weeks after restarting ART in the STI group. To evaluate HIV-induced CD8^+^ T-cell responses, additional blood specimens were collected in both groups at the beginning of an STI cycle and when ART was restarted in the STI group.

### 2.3. HIV Peptides and Preparation of Pools

Uganda is predominantly infected with HIV-1 clades A and D as well as recombinants of these [18, 39–41]. We therefore attempted to work with peptide pools that were matching these strains as much as possible. Peptides were obtained through the National Institute of Health, AIDS Research Reference Reagent programe (https://www.aidsreagent.org/Index.cfm). Unfortunately, peptides available from this source were only representative of clade B with the exception of peptides corresponding to the Gag region which matched the strains found in our study population. Individual peptides consisted of 20-mer peptides overlapping by 11 amino acids and spanning the HIV-1 consensus clade A (92UG037) and D (94UG114) Gag sequences, as well as 15-mer peptides overlapping by 11 amino acids spanning the HIV-1 clade B Nef, Tat, Vif, Rev, Vpr, Vpu, and Pol consensus sequences. Individual peptides were grouped together into pools according to HIV protein. Each individual peptide within a pool was used at a final concentration of 2 *μ*g/ml. Due to limitations in blood volume, it was not possible to map individual responding T-cell epitopes; consequently, CD8^+^ T-cell responses to complete HIV-1 protein pools are presented.

### 2.4. Intracellular Cytokine Staining Procedure

Activation and processing of peripheral blood mononuclear cells (PBMCs) for intracellular cytokine staining analysis was performed as previously described [42]. Briefly, activation reagents (HIV peptide pools and 1 *μ*g/mL costimulatory CD28 and CD49d antibodies (BD Biosciences)) were added to 1 mL of fresh whole heparinised blood and incubated at 37°C, in a 5% CO_2_ in air atmosphere for 1 hour, followed by further 5 hours in the presence of a secretion inhibitor (Golgi Plug, 10 *μ*g/ml, BD Biosciences). Red blood cells were subsequently lysed with FACS lysis solution (BD Biosciences). The PBMCs were fixed and permeabilised with FACS permeabilising buffer (BD Biosciences) according to the manufacturer's protocol and then simultaneously stained for 1 hour in the dark with surface antibodies CD3-FITC, CD8^+^-PerCP and intracellular antibodies IFN-*γ*-APC and Perforin-PE (BD Biosciences) at room temperature. Stained cells were washed and fixed with Cellfix (BD Biosciences). At least 200,000 PBMCs were acquired on a FACSCalibur flow cytometer (BD Biosciences). Negative controls (backgrounds) were autologous PBMCs that were not stimulated with peptides but had otherwise been treated identically. Positive controls were specimens that had been stimulated with 10 *μ*L of 1 *μ*g/*μ*L of Staphylococcal Enterotoxin B (SEB). Flow cytometry data was analysed using CellQuest (BD Biosciences). Criterion for evaluating positive responses was ≥0.03% of CD8^+^ T cells responding to any of the HIV peptide pools after subtracting the background cytokine production. Response to the two Gag pools (clade A and D) was evaluated collectively as the mean Gag response. Monofunctional T cells were defined as those secreting either IFN-*γ* or Perforin alone; bifunctional T cells were defined as cells that simultaneously released both Perforin and IFN-*γ*. Breadth of response was defined as the number of HIV protein pools targeted by each participant. The frequency of HIV-induced response was defined as the proportion (%) of CD8^+^ T cells inducing HIV-specific IFN-*γ* or Perforin responses or both. The frequency of responders was defined as the proportion of participants in which HIV-specific T-cell responses were induced. 

### 2.5. Statistical Analysis

Medians and interquartile ranges (IQR) were used for all summary presentations of CD4^+^ T-cell counts and T-cell responses. Kruskal-Wallis rank and Mann-Whitney tests were used to compare medians. Proportions were compared using Pearson's chi-square test. Median alterations in response for each patient were compared by evaluating the increase or decrease in CD8^+^ T-cell response at the beginning and end of a 12-week cycle. This approach provided a more powerful test of differences between CT and STI, since it allowed each patient to act as their own control therefore reducing variability. Graph Pad 5.0 and Excel were used for graphical presentations of the data. All statistical analyses were performed using Stata v8.0 (Stata Corp, Texas).

## 3. Results

### 3.1. Study Population and Baseline Characteristics

Of the 120 participants recruited into this substudy, 88 subjects (42 STI and 46 CT) had completed one cycle of STI by the time the Trial Steering Committee (TSC) followed the recommendation of the DSMC to terminate the STI trial due to observed inferior clinical outcomes among participants randomized to the STI group. Similar to the overall demographics seen in the main DART trial, our study evaluated significantly more females (*n* = 60) than males (*n* = 28). There was no significant difference in the median age of CT (38; 33–44 years) and STI (38; 32–44 years) subjects. The proportion of STI and CT subjects with pre-STI/CT randomization ART exposure duration of either 52 (28/46 versus 22/42, resp.) or 76 weeks (18/46 versus 20/42, resp.) was also comparable.

### 3.2. Comparison of CD4^+^ T-Cell Counts

In the current study subjects, the median CD4^+^ counts at ART initiation within the main DART trial were comparable between STI (124; IQR 87–177 CD4^+^ T cells/*μ*L) and CT subjects (129; IQR 77–159 CD4^+^ T cells/*μ*L). Inline with the findings of the main DART STI trial [38], we did not find any difference between CD4^+^ T-cell counts of STI (391; 334–449 cells/*μ*L) and CT subjects (391; 333–449 cells/*μ*L) at STI/CT randomization. Additionally, CD4^+^ T-cell counts were comparable among subjects that were randomized after 52 weeks (397; 334–457 cells/*μ*L) or 76 weeks of ART initiation (385; 330–434 cells/*μ*L). These data suggest that an additional 24 weeks of ART exposure prior to STI/CT randomization did not significantly influence the CD4^+^ T-cell counts anymore at this stage.

### 3.3. Frequency of HIV-Specific CD8^+^ T-Cell Responses at STI/CT Randomization

**Figure 1:**
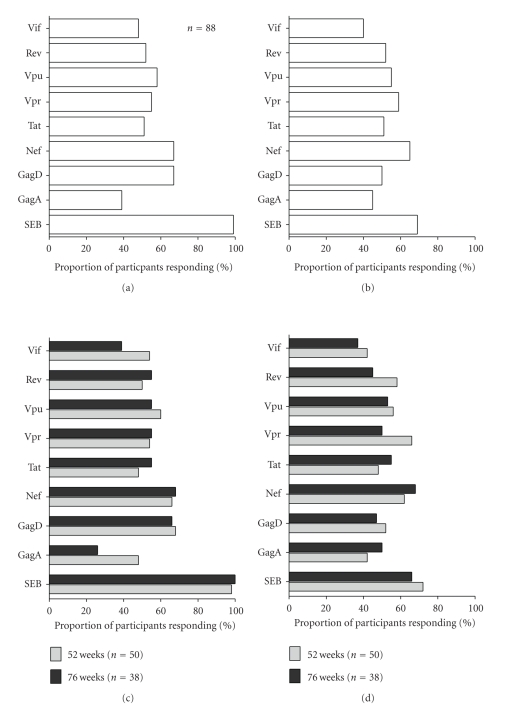
HIV-specific CD8^+^ T-cell responses at STI/CT randomization. This figure compares the proportions (%) of participants (*n* = 88) inducing (a) HIV-specific IFN-*γ* or (b) Perforin responses at the time point of STI/CT randomization and proportions of participants inducing (c) HIV-specific IFN-*γ* or (d) Perforin responses at STI/CT randomization initiated 52 or 76 weeks after ART.

We evaluated the frequency of HIV Gag (clade A and D), Nef, Tat, Vif, Rev, Vpr, and Vpu (all clade B)-induced T-cell responses as the proportion of subjects with detectable virus-specific CD8^+^ T-cell IFN-*γ* or Perforin. Overall, HIV-specific CD8^+^ T-cell responses were detected against all the seven evaluated HIV proteins as follows: Gag- (85%), Nef- (67%), Tat- (51%), Vpr- (53%), Vpu- (58%), Rev- (52%) and Vif- (48%). At STI/CT randomization, 99% of the participants had the potential to induce IFN-*γ* while 31% completely lacked the intrinsic potential to induce Perforin (as evaluated by stimulation with SEB) (Figures 1(a) and 1(b), resp.) 

We then evaluated the relationship between the duration of ART uptake before STI/CT randomization, CD4^+^ count at ART initiation and the frequency of virus-specific CD8^+^ T-cell responses at STI/CT randomization. Both HIV-specific IFN-*γ* (Figure 1(c)) and Perforin T-cell responses (Figure 1(d)) did not significantly differ between participants who received ART for 52 or 76 weeks. Similarly, there was no correlation between the CD4^+^ count at ART initiation and the frequency of virus-induced T-cell responses (data not shown). Taken together, these findings suggest that the additional 24 weeks of ART in individuals who underwent STI/CT randomization at 76 weeks did not significantly modify the proportion of subjects with detectable HIV-induced CD8^+^ T-cell recognition.

### 3.4. Breadth of HIV-Induced CD8^+^ T-Cell Responses at STI/CT Randomization

**Figure 2:**
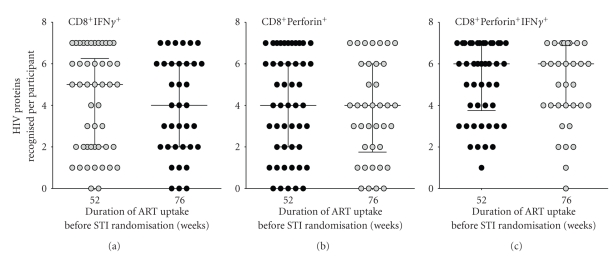
Relationship between breadth of HIV-specific CD8^+^ T-cell recognition at STI/CT randomization and the duration of pre-ART randomization. This figure evaluates whether ART uptake for either 52 weeks or 76 weeks preceding randomization had any influence on the breadths of HIV-specific (a) CD8^+^IFN*γ* ^*+*^, (b) CD8^+^Perforin^*+*^, or (c) CD8^+^IFN*γ* ^+^Perforin^*+*^ T-cell responses. Individual HIV peptides were grouped together in pools according to HIV protein. HIV-specific T-cell recognition was evaluated against these pools that were based on consensus sequences of HIV-1 Gag clades A (92UG037) and D (94UG114); and consensus sequences of HIV-1 clade B (Nef, Tat, Vif, Rev, Vpr, Vpu, and Pol). HIV-specific responses to Gag (clades A and D) were considered concomitantly. Breadth was defined as the number of HIV protein pools recognised per individual. Horizontal bars represent medians and interquartile ranges.

Breadth of response was defined as the number of HIV protein pools targeted by each subject. Overall, breadths of CD8^+^IFN*γ* ^+^, CD8^+^Perforin^+^ and CD8^+^IFN*γ* ^+^Perforin^+^ at STI/CT randomization lacked correlation with the nadir CD4^+^ count and did not differ in subjects that took ART for either 52 or 76 weeks before STI/CT randomization (Figure 2). These data suggest that the additional 24 weeks of ART uptake before STI randomization did not influence the breadth of virus-specific T-cell responses.

### 3.5. Magnitude of HIV-Induced T-Cell Responses at STI/CT Randomization

**Figure 3:**
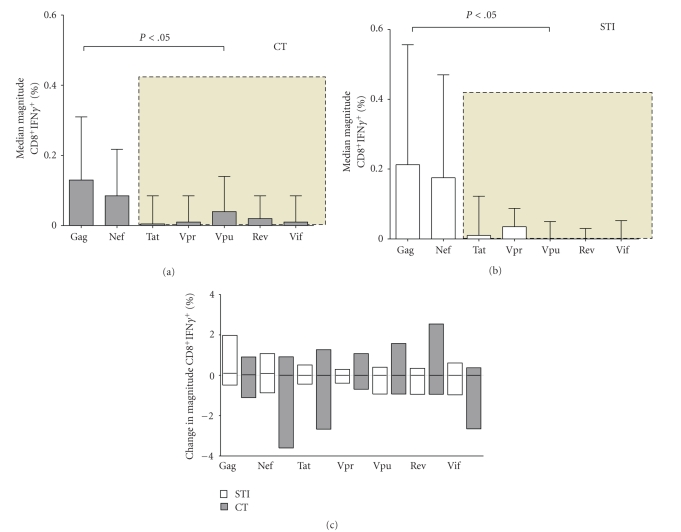
Magnitudes of CD8^+^ T-cell responses associated with STI. This figure compares the median magnitudes of HIV-induced CD8^+^ T-cell IFN-*γ* responses in (a) STI and CT participants after one cycle of STI or matching time on CT. Bars represent medians, while error bars represent interquartile ranges. The shaded areas represent HIV proteins that induced significantly lower magnitudes of CD8^+^IFN-*γ* ^+^ than Gag or Nef. (c) illustrates the median change in magnitudes of HIV-specific IFN-*γ* responses after one cycle of STI or matching time on CT. Horizontal bars represent medians.

Magnitude of T-cell response was defined as the proportion (%) of CD3^+^CD8^+^ T cells inducing release of either IFN-*γ*, Perforin or both, following stimulation with HIV-1 Gag, Nef, Tat, Vpr, Vpu, Rev and Vif peptide pools. Gag data is presented as the average of the two Gag pools. At STI/CT randomization, the inherent magnitude of T-cell responses as evaluated using Staphylococcal Enterotoxin B (SEB) was significantly lower for Perforin (0; 0–0.12% CD3^+^CD8^+^ T cells) compared to IFN-*γ* (5; 3–9% CD3^+^CD8^+^ T cells), respectively; *P* < .0001, Mann-Whitney test. Thus, it may be seen that HIV-1-specific CD8^+^ T-cell responses in this cohort were mostly comprised of IFN-*γ*-secreting cells at the time point of STI/CT randomization. We therefore used the IFN-*γ* data to evaluate the relationship between STI and the pattern of virus-specific T-cell recognition. Overall, Nef and Gag (mean of the two Gag pools) induced significantly higher magnitude of CD8^+^ T-cell responses than Tat, Vpr, Vpu, Rev, and Vif in both CT and STI participants; magnitudes of Gag- and Nef-induced responses did not significantly differ (Figures 3(a) and 3(b)).

### 3.6. Magnitudes after One STI Cycle (12-Week On/12-Week Off)

The median change in Nef-, Tat-, Vpr-, Vpu-, Rev-, and Vif-induced IFN-*γ* magnitudes remained comparable among CT and STI subjects although there was significant increase in magnitude of Gag-induced IFN-*γ* (Figure 3(c)). Despite the apparent improvement in magnitudes of Gag-induced IFN-*γ* in STI subjects, the magnitude of Perforin responses remained significantly lower compared to IFN-*γ* even after 12 weeks of CT or STI, and the ability (proportion of individuals) of the STI arm to induce simultaneously release of both IFN-*γ* and Perforin did not improve even for Gag (data not shown). Taken together, these data suggest a preexisting functional impairment of CD8^+^ T cells in this cohort, mainly characterised by diminution of Perforin-inducing potential, which remained unchanged after 12 weeks of STI.

### 3.7. Breadths after One STI Cycle (12-Week On/12-Week Off)

**Figure 4:**
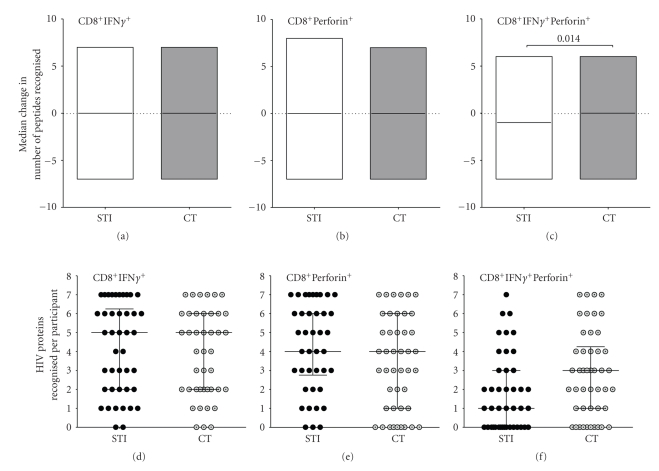
Breadths of CD8^+^ T-cell responses after 12 weeks of STI and matching time point on CT. Study participants were evaluated for CD8^+^ T-cell responses to complete peptide pools corresponding to Gag, Nef, Tat, Vpr, Vpu, Rev and Vif HIV proteins. Response to the two Gag pools was analysed concomitantly to represent response to the Gag protein. Breadth of CD8^+^ T-cell response was defined as the number of HIV pools recognised by an individual. Median change in breadth was defined as the increase or decrease in number of pools recognised. Positive readouts indicate increase while negative readouts indicate decrease. The figure demonstrates changes in the breadth of CD8^+^IFN*γ* ^+^ (a), CD8^+^Perforin^+^ (b) and CD8^+^IFN*γ* ^+^Perforin^+^, (c) responses among CT and STI participants; and compares the median number of peptide pools targeted with the induction of CD8^+^IFN*γ* ^+^ (d), CD8^+^Perforin^+^ (e), and CD8^+^IFN*γ* ^+^Perforin^+^ (f) at the end of one STI cycle. Horizontal lines represent medians.

We evaluated the relationship between one STI cycle and the breadth of HIV-induced CD8^+^ T-cell responses (number of HIV proteins recognised per subject). Recognition of the two Gag pools was analyzed concomitantly to represent the average response to the Gag protein. Median change in breadth was defined as the increase or decrease in number of HIV pools recognised after completing one STI cycle. After 12 weeks of STI or CT, the median change in breadth of CD8^+^IFN*γ* ^+^ (Figure 4(a)) and CD8^+^Perforin^+^ (Figure 4(b)) did not significantly differ between STI and CT participants. However, STI was associated with a significant reduction in breadth of bifunctional CD8^+^IFN*γ* ^−^Perforin^+^ responses (median −1, IQR 0 to −3.3 protein pools) compared to CT, (median 0, IQR −2.0 to 3.0 HIV protein pools) (Figure 4(c)). Similarly, there was no difference in the breadth of monofunctional IFN-*γ* responses targeted (Figures 4(d) and 4(e)). However, 12 weeks of STI resulted in significantly lower bifunctional T-cell responses, (median 1, IQR 0–3 protein pools) compared to a similar timeframe on continuous treatment (median 3, IQR 1–4 protein pools); *P* = .027 (Figure 4(f)), Mann-Whitney test. These data suggest that STI was associated with degeneration rather than restoration of functional CD8^+^ T-cell responses in this cohort.

## 4. Discussion

Better understanding of HIV-induced CD8^+^ T-cell responses in chronic HIV-1 infection may be important for the development of preventive or therapeutic approaches designed to enhance T-cell-mediated immunity. In this study, we used structured treatment interruption that was initiated within the DART trial [38] as a model to reevaluate the hypothesis that STI would allow for regeneration of HIV-specific responses through cyclical viral antigen exposure. The DART cohort differed from previous studies that assessed individuals initiated on ART at an earlier HIV disease state [8, 10, 43]. The principle findings of this study were firstly that magnitudes of CD8^+^ T-cell responses did not significantly differ following STI or CT for an identical timeframe, secondly that breadths of monofunctional CD8^+^ T-cell responses were comparable between STI and CT, and thirdly that STI was apparently associated with significant loss of bifunctional CD8^+^ T-cell responses.

Because the overall potential to induce Perforin was apparently impaired in this cohort initiated on ART at around 125 (85–173) CD4^+^ T cells/*μ*L, we used the IFN-*γ* data to evaluate the relationship between STI and the profile of CD8^+^ T-cell responses. Overall, Gag and Nef induced significantly higher magnitude of IFN-*γ* responses than other HIV proteins tested at STI/CT randomization in both STI and CT participants; this finding was consistent with others that reported relative immunodominance of Gag and Nef in this and other populations [17, 18]. However, there were some limitations related to the reagents used to evaluate CD8^+^ T-cell responses, and the Gag results need to be interpreted with caution. The peptides used to stimulate CD8^+^ T cells matched the infecting HIV strains only in the Gag region, whereas clade B peptides were used to evaluate responses to the other proteins. Consequently, virus-specific CD8^+^ T-cell responses to other HIV proteins may not have been detected as efficiently as responses to Gag, where the infecting viral sequences were better represented. Indeed, several studies have shown that interclade cross-reactivity response rates tend to be lower than clade-specific responses [18, 44, 45] and that use of variant sequences can result in reduced T-cell recognition. 

HIV-specific T-cell responses are common in infected adults, become progressively dysfunctional during chronic virus persistence, and exhibit rapid decay during ART uptake [1, 46–48]. Previous studies suggested that several functional attributes of HIV-specific CD8^+^ T cells influence the differential disease outcome in chronic infection [34–36, 49, 50]. Continuous use of potent ART has been shown to significantly suppress viral replication [51–53], thereby potentially allowing for limited T-cell functional restoration. In this cohort, STI significantly correlated with diminution of CD8^+^IFN*γ* ^+^Perforin^+^ bifunctional T cells. This was consistent with previous reports of STI-associated functional impairment of T cells [19]; attribution of the prognostic outcomes of STI to a defined virus-specific CD8^+^ T-cell functional profile [50] and to findings that indicated that continued use of potent ART could defer this impairment [46, 54].

In this study, an extra 24 weeks of therapy in individuals that were randomized to STI or CT at 76 weeks did not result in any apparent change in profile of HIV-induced CD8^+^ T-cell responses when compared to those that were randomized at 52 weeks. Consequently, these data suggest that the observed difference in breadth of the bifunctional CD8^+^ T cell responses may be possibly attributed to STI rather than to the restoration of functionality by continued treatment. These data collectively suggest an STI-associated impairment of CD8^+^ T-cell functionality and that the quality rather than the quantity of the HIV-specific CD8^+^ T-cell responses correlated with the dynamics of STI.

There were other limitations to this study. First of all, we evaluated a population that was initiated onto ART at a very advanced stage of HIV disease potentially masking possible positive effect that others observed in individuals who initiated ART much earlier in disease [55, 56]. The benefits and risks of antiretroviral therapy can vary considerably with stage of disease, mainly due to irreversible destruction of the immune system that occurs as HIV infection progresses. Treatment during acute HIV infection may preserve and reconstitute HIV-specific immune function. Alternatively, the primary goal of late-stage disease treatment is to control viral replication leading to decreased morbidity and increased survival. While our sample size was not big enough to evaluate the effect of disease stage, exploration of the data revealed no correlation between the CD8^+^ T-cell responses and the original CD4^+^ count before ART initiation. Secondly, we evaluated only one cycle of STI; consequently, we could not extrapolate on possible longer-term effects of STI that have been reported in studies that used a longitudinal approach to evaluate several STI cycles [31]. Lastly, this study was limited to collect 10 mls of blood from each subject at each study visit. Subsequently, individual peptides were grouped according to HIV protein and evaluated as pools. As a result, it was not possible to map individual responding T-cell epitopes in order to determine whether it was responses specific for a particular epitope that disappeared during an STI or all epitopes were affected equally.

In summary, the current study found no evidence to support the hypothesis that STI would enhance regeneration of HIV-induced T-cell responses in individuals starting ART with advanced HIV disease state. Instead, our findings are inline with previous reports that indicated ART-associated improvement of CD8^+^ T-cell functional markers [52, 57], preferential destruction of HIV-infected CD4^+^ T cells during treatment interruptions [58], and viral burden-induced deterioration of the quality of CD8^+^ T-cell response even when HIV-specific IFN-*γ* responses were still apparent [59]. This study found no evidence to support STI as a strategy for ART delivery in African patients starting therapy with CD4^+^ counts <200 cells/*μ*L.

##  Disclaimer

This manuscript has not been published in its current form or a substantially similar form. There are no financial, consultant, institutional and other relationships that might lead to bias or a conflict of interest.
